# Laminoplasty versus laminectomy and fusion for multilevel cervical compressive myelopathy: A meta-analysis

**DOI:** 10.1097/MD.0000000000003588

**Published:** 2016-06-10

**Authors:** Feng-Yu Liu, Si-Dong Yang, Li-Shuang Huo, Tao Wang, Da-Long Yang, Wen-Yuan Ding

**Affiliations:** a Department of Spinal Surgery, The Third Hospital of Hebei Medical University; b Hebei Provincial Key Laboratory of Orthopedic Biomechanics; c Department of Endocrinology, The Second Hospital of Hebei Medical University, Shijiazhuang, China.

**Keywords:** cervical, fusion, laminectomy, laminoplasty, meta-analysis

## Abstract

This is a meta-analysis to compare the results between laminoplasty and laminectomy followed by fusion for the patients with multilevel cervical compressive myelopathy. An extensive search of literature was performed in MEDLINE, Embase, the Cochrane library, CNKI, and WANFANG. The following outcome measures were extracted: the Japanese Orthopaedic Association (JOA) scores, cervical curvature index (CCI), visual analog scale (VAS), cervical lordosis (C2–7), complications, blood loss, and operation time. Data analysis was conducted with RevMan 5.3 and STATA 12.0. A total of 23 studies comprising 774 and 743 patients treated with laminoplasty and laminectomy followed by fusion, respectively, were included in the final analysis. The pooled analysis showed that there was no significant difference in preoperative JOA scores [*P* = 0.89], postoperative JOA scores [*P* = 0.13], JOA scores improvement rate [*P* = 0.27], preoperative CCI [*P* = 0.15], postoperative CCI [*P* = 0.14], preoperative VAS [*P* = 0.41], postoperative VAS [*P* = 0.52], preoperative cervical lordosis (C2–7) [*P* = 0.46], postoperative cervical lordosis (C2–7) [*P* = 0.67], total complications [*P* = 0.07], axial pain [*P* = 0.94], and blood loss [*P* = 0.51]. However, there were significant difference in operation time (WMD = −19.57 [−32.11, −7.02], *P* = 0.002) and C5 palsy (OR = 0.26 [0.15, 0.44], *P* < 0.001). As compared with laminectomy followed by fusion, expansive laminoplasty showed no significant differences in JOA scores, CCI, ROM, VAS, cervical lordosis (C2–7), axial pain, total complications, and blood loss, but shorter operation time and fewer C5 palsy.

## 1 Introduction

Multilevel cervical compressive myelopathy is a clinically symptomatic condition usually caused by multisegment cervical spondylotic myelopathy, congenital cervical canal stenosis, or ossification of the posterior longitudinal ligament (OPLL).^[1]^ Myelopathy usually lead to progressive and stepwise deterioration of neurologic function. If the symptoms do not respond to conservative treatment, surgical treatment should be considered. Surgical treatment with either anterior or posterior approaches can result in satisfactory clinical results. When ≥3 segments are involved, the complication rates associated with anterior surgery accelerate. It makes posterior options more attractive.^[2]^

The posterior procedures, including laminoplasty, laminectomy alone, and laminectomy followed by fusion, are recognized as a reliable and effective way in treating multilevel cervical compressive myelopathy. Laminectomy was initially regarded as the gold standard treatment of multilevel cervical myelopathy due to the extensive decompression. But the technique is associated with many drawbacks, especially postoperative segmental instability and kyphosis.^[3]^ Laminectomy followed by fusion addressed these drawbacks. Laminoplasty is developed in Japan and allowed extensive cord decompression while preserving motion with less substantial alteration to the natural biomechanics of the cervical spine.^[4]^

Some studies show that laminoplasty is superior to laminectomy followed by fusion. Other studies show opposite results. There is no clear conclusion on which method, laminoplasty or laminectomy with fusion, is better. Therefore, we performed a meta-analysis to assess the effectiveness and safety of these 2 surgical procedures for multilevel cervical compressive myelopathy.

## 2 Materials and methods

### 2.1 Ethics statement

There is no need to seek consent from patients, as in this study all the data were collected and analyzed anonymously without any potential harm to the patients; this is approved by Ethics Committee of The Third Hospital of Hebei Medical University.

### 2.2 Search methods and selection of studies

An extensive search of literature was performed in MEDLINE, Embase, the Cochrane library, CNKI, and WANFANG. It was not restricted to year of publication and language was restricted to Chinese or English. The following key words were used for search: “cervical spondylotic myelopathy,” “CSM,” “ossification of posterior longitudinal ligament,” “OPLL,” “laminoplasty,” “laminectomy,” and “fusion,” with various combinations of the operators “AND,” “NOT,” and “OR.”

### 2.3 Inclusion criteria

Studies were included if they met the following criteria: (1) randomized or nonrandomized controlled study; (2) included patients with cervical spondylotic myelopathy, cervical canal stenosis, or ossification of posterior longitudinal ligament; (3) included patients who underwent posterior decompression surgery; (4) laminoplasty and laminectomy followed by fusion were compared.

### 2.4 Selection of studies

Two reviewers (Feng-Yu Liu and Si-Dong Yang) independently reviewed all subjects, abstracts, and the full text of articles. Then the eligible trials were selected according to the inclusion criteria. When consensus could not be reached, a third reviewer (Wen-Yuan Ding) was consulted to resolve the disagreement.

### 2.5 Data extraction and management

Two reviewers (Feng-Yu Liu and Si-Dong Yang) extracted data independently. The data extracted included the following categories: study ID, study design, study location, patients (diagnoses, age, sex), and clinical outcomes (JOA, CCI, VAS, cervical lordosis [C2–7], complications, blood loss, and operation time).

### 2.6 Statistical analysis

As for Data analysis, we used 2 regular software: RevMan 5.3 (The Nordic Cochrane Center, The Cochrane Collaboration, Copenhagen, Denmark) and STATA 12.0 (Stata Corporation, College Station, TX). We used risk ratio (RR), which is a summary statistic, to analyze dichotomous variables, and the standardized mean difference (WMD) to analyze continuous variables. Both were reported with 95% confidence intervals (CIs), and a *P* < 0.05 was used as the level of statistical significance. We used random-effects or fixed-effects models, which were depended on the heterogeneity of the studies included. We use *I*^2^ to test heterogeneity, where *I*^2^ > 50% implied heterogeneity.

## 3 Results

### 3.1 Search results

**Figure 1:**
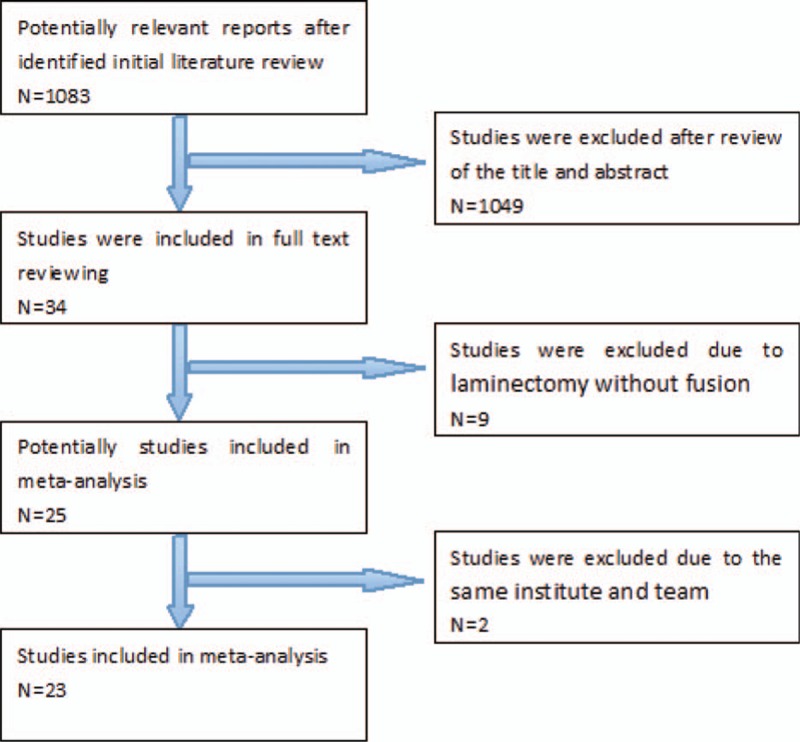
Flow diagram of study selection.

The database search resulted in 418 studies in MEDLINE, 383 studies in Embase, 8 studies in the Cochrane Library, 177 in WANFANG, and 97 in CNKI. Of these, 1049 were excluded for not being comparative studies, not human studies, unrelated to the topic at hand, or being review articles or case reports after review of the abstract and title. Another 9 articles were excluded because of laminectomy without fusion. Two studies studied at the same institute and team, the cases maybe overlapped, and we selected 1 article. As a result, a total of 23 studies were used for this meta-analysis. The literature search procedure was shown in Fig. 1.

### 3.2 Baseline characteristics and quality assessment

A total of 23 studies comprising 774 and 743 patients treated with laminoplasty and laminectomy followed by fusion, respectively, were included in the final analysis. There were no significant differences between groups with regards to age, sex, and follow-up. Table 1 presents the baseline characteristics of the 2 groups.

**Table 1:**
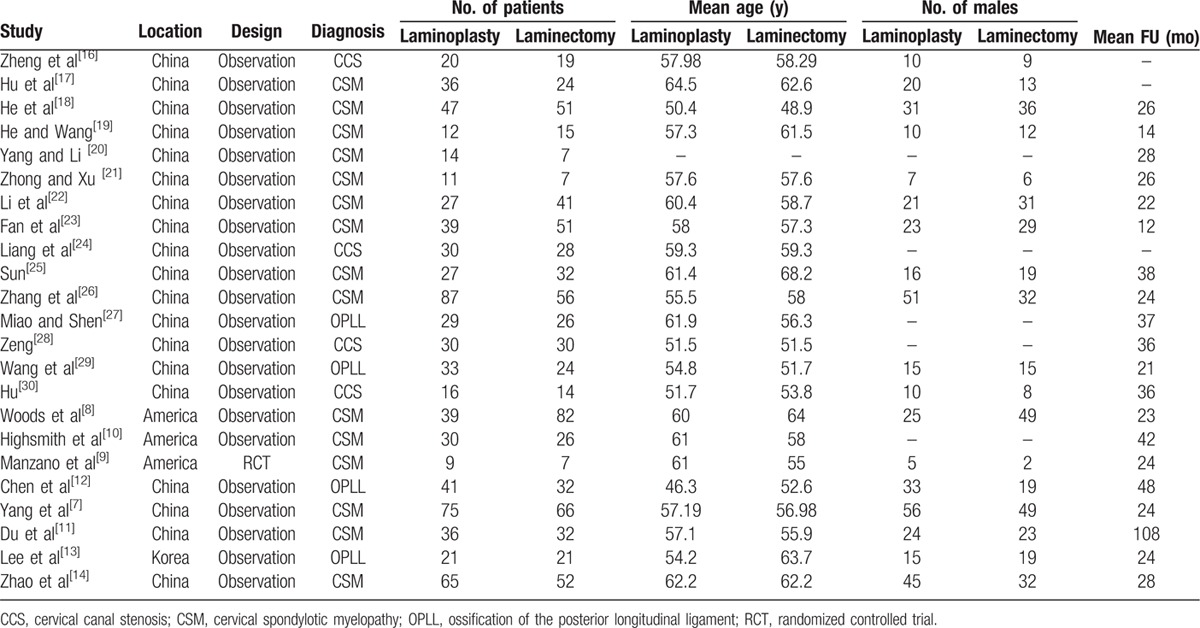
Characteristics of included studies.

To assess the quality of each study, we used the Newcastle Ottawa Quality Assessment Scale (NOQAS). This scale for nonrandomized case controlled studies and cohort studies had a maximum of 9 points, which included the quality of selection, comparability, exposure, and outcomes for study participants. Of these studies, 18 scored 8 points and 5 scored 7 points. Therefore, the quality of each study was relatively high (Table 2).

**Table 2:**
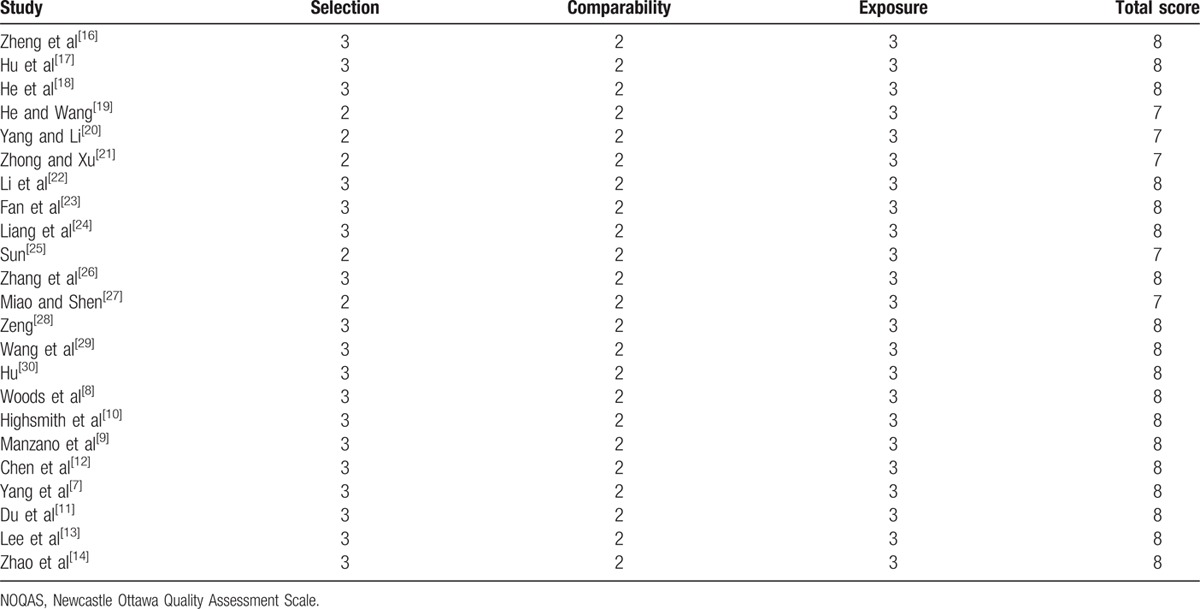
The quality assessment according to the NOQAS of each study.

### 3.3 Clinical outcome

**Figure 2:**
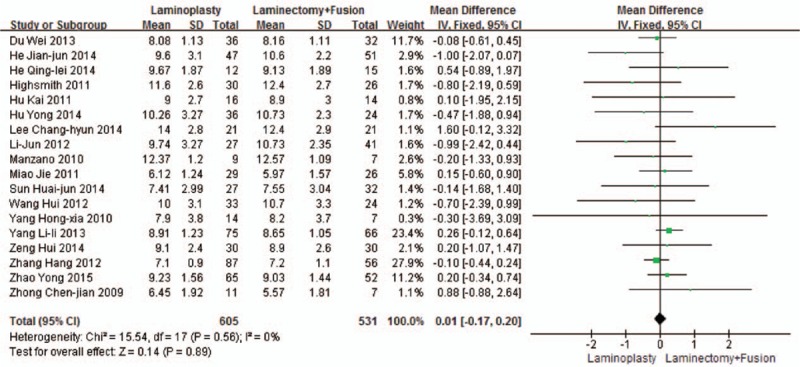
The standardized mean difference (SMD) estimate for the preoperative JOA score. JOA, Japanese Orthopaedic Association.

**Figure 3:**
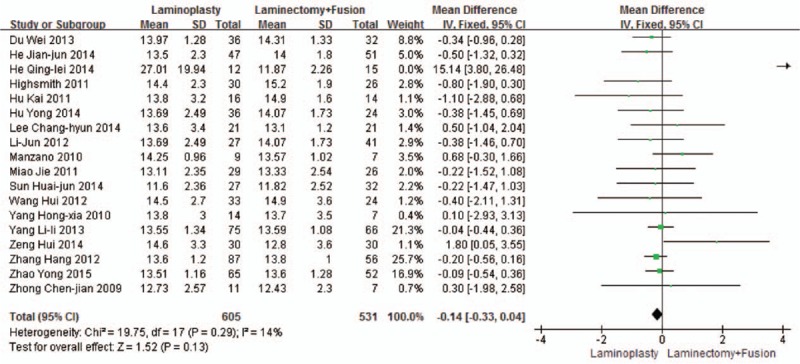
The standardized mean difference (SMD) estimate for the postoperative JOA score. JOA, Japanese Orthopaedic Association.

Eighteen studies reported the JOA scores (n = 605 in the laminoplasty group and 531 in the laminectomy + fusion group). Preoperative JOA scores were similar between the 2 groups (*P* = 0.89, WMD = 0.01 [–0.17, 0.20]; heterogeneity: *P* = 0.56, *I*^2^ = 0%, fixed-effect model, Fig. 2). Postoperative JOA scores were similar between the 2 groups (*P* = 0.13, WMD = –0.14 [–0.33, 0.04]; heterogeneity: *P* = 0.29, *I*^2^ = 14%, fixed -effect model, Fig. 3).

**Figure 4:**
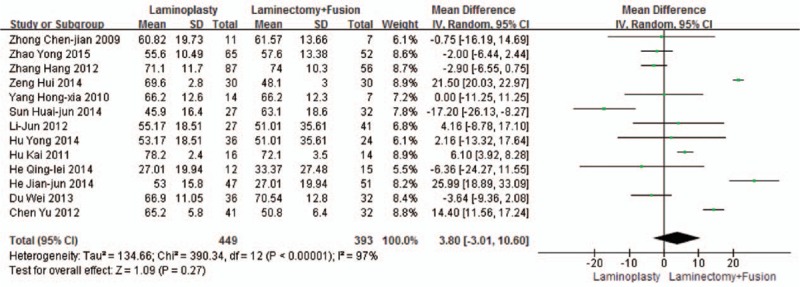
The standardized mean difference (SMD) estimate for the JOA scores’ improvement rate. JOA, Japanese Orthopaedic Association.

Thirteen studies reported the JOA scores improvement rate (n = 449 in the laminoplasty group and 393 in the laminectomy + fusion group). JOA scores’ improvement rate was similar between the 2 groups (*P* = 0.27, WMD = 3.80 [–3.01, 10.60]; heterogeneity: *P* < 0.001, *I*^2^ = 97%, random-effect model, Fig. 4).

**Figure 5:**
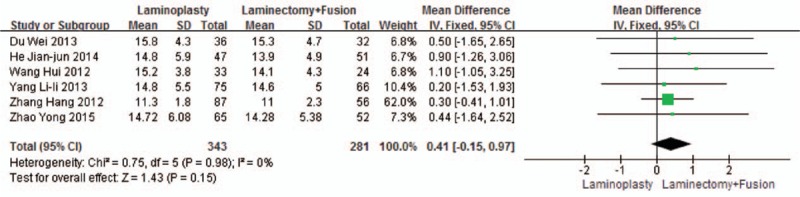
The standardized mean difference (SMD) estimate for preoperative CCI. CCI, cervical curvature index.

**Figure 6:**
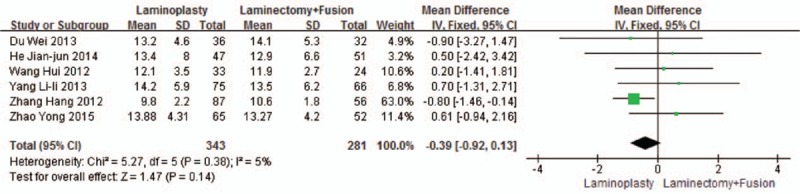
The standardized mean difference (SMD) estimate for postoperative CCI. CCI, cervical curvature index.

Six studies reported the CCI (n = 343 in the laminoplasty group and 281 in the laminectomy + fusion group). Preoperative CCI was similar between the 2 groups (*P* = 0.15, WMD = 0.41 [−0.15, 0.97]; heterogeneity: *P* = 0.98, *I*^2^ = 0%, fixed-effect model, Fig. 5). Postoperative CCI was similar between the 2 groups (*P* = 0.14, WMD = −0.39 [−0.92, 0.13]; heterogeneity: *P* = 0.38, *I*^2^ = 5%, fixed-effect model, Fig. 6).

**Figure 7:**
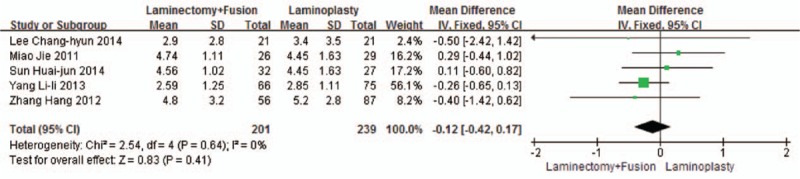
The standardized mean difference (SMD) estimate for preoperative VAS. VAS, visual analog scale.

**Figure 8:**
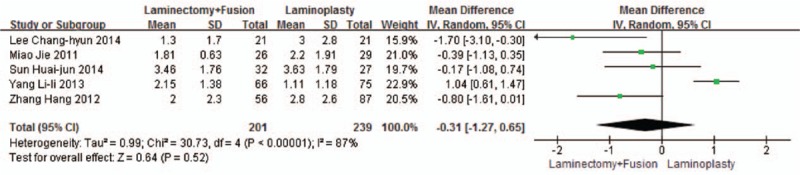
The standardized mean difference (SMD) estimate for postoperative VAS. VAS, visual analog scale.

Five studies reported the VAS (n = 239 in the laminoplasty group and 201 in the laminectomy + fusion group). Preoperative VAS was similar between the 2 groups (*P* = 0.41, WMD = 0.12 [−0.17, 0.42]; heterogeneity: *P* = 0.64, *I*^2^ = 0%, fixed-effect model, Fig. 7). Postoperative VAS was similar between the 2 groups (*P* = 0.52, WMD = 0.31 [−0.65, 1.27]; heterogeneity: *P* < 0.001, *I*^2^ = 87%, random-effect model, Fig. 8).

**Figure 9:**
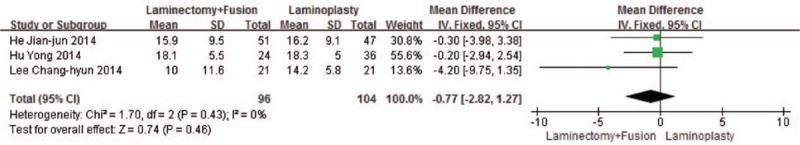
The standardized mean difference (SMD) estimate for preoperative cervical lordosis (C2–7).

**Figure 10:**
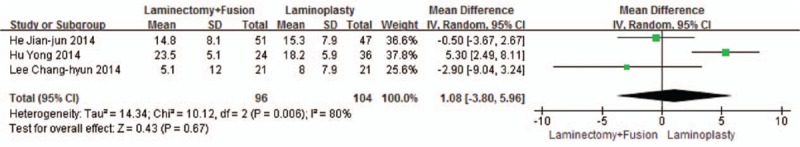
The standardized mean difference (SMD) estimate for postoperative cervical lordosis (C2–7).

Three studies reported the cervical lordosis (C2–7) (n = 104 in the laminoplasty group and 96 in the laminectomy + fusion group). Preoperative cervical lordosis (C2–7) was similar between the 2 groups (*P* = 0.46, WMD = 0.77 [–1.27, 2.82]; heterogeneity: *P* = 0.43, *I*^2^ = 00%, fixed-effect model, Fig. 9). Postoperative cervical lordosis (C2–7) was similar between the 2 groups (*P* = 0.67, WMD = –1.08 [–5.96, 3.80]; heterogeneity: *P* = 0.006, *I*^2^ = 80%, random-effect model, Fig. 10).

### 3.4 Complications

**Figure 11:**
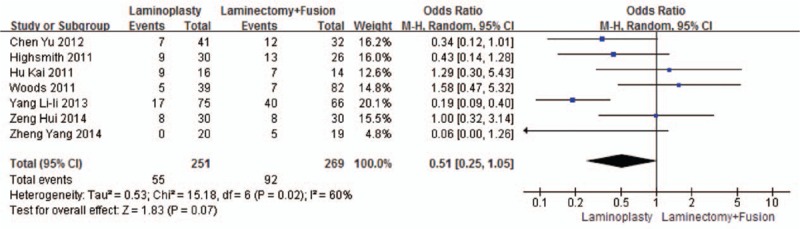
The odds ratio (OR) estimate for total complications.

Seven studies reported the total complications (n = 251 in the laminoplasty group and 269 in the laminectomy + fusion group). Total complications were similar between the 2 groups (*P* = 0.07, OR = 0.51 [0.25, 1.05]; heterogeneity: *P* = 0.02, *I*^2^ = 60%, random-effect model, Fig. 11).

**Figure 12:**
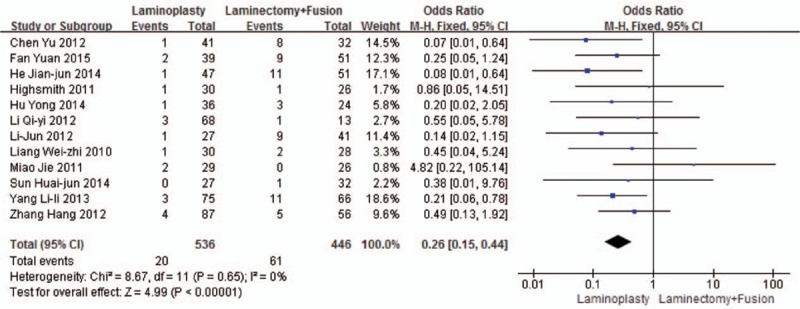
The odds ratio (OR) estimate for the C5 palsy.

Twelve studies reported the C5 palsy (n = 536 in the laminoplasty group, and 446 in the laminectomy + fusion group). The laminoplasty group showed fewer C5 palsy, compared with the laminectomy + fusion group (*P* < 0.001, OR = 0.26 [0.15, 0.44]; heterogeneity: *P* = 0.65, *I*^2^ = 0%, fixed-effect model, Fig. 12).

**Figure 13:**
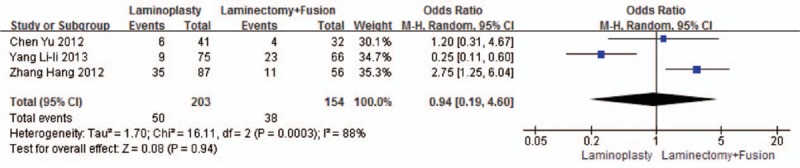
The odds ratio (OR) estimate for the axial pain.

Three studies reported the axial pain (n = 203 in the laminoplasty group and 154 in the laminectomy + fusion group). Axial pain was similar between the 2 groups (*P* = 0.94, OR = 0.94 [0.19, 4.60]; heterogeneity: *P* < 0.001, *I*^2^ = 88%, random -effect model, Fig. 13).

### 3.5 Blood loss and operation time

**Figure 14:**
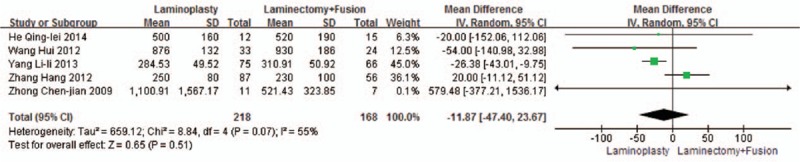
The standardized mean difference (SMD) estimate for blood loss.

Five studies reported the intraoperative blood loss (n = 218 in the laminoplasty group, and 168 in the laminectomy + fusion group). Blood loss was similar between the 2 groups (P = 0.51, WMD = −11.87 [−47.40, 23.67]; heterogeneity: *P* = 0.07, *I*^2^ = 55%, random-effect model, Fig. 14).

**Figure 15:**
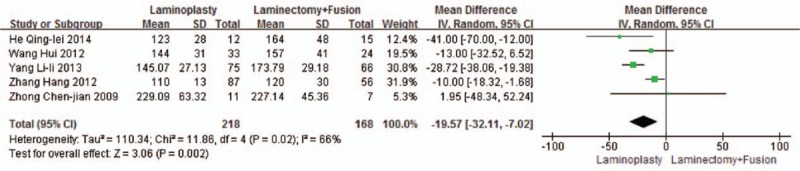
The standardized mean difference (SMD) estimate for operation time.

Five studies reported the operation time (n = 218 in the laminoplasty group and 168 in the laminectomy + fusion group). The laminoplasty group showed shorter operation time, compared with the laminectomy + fusion group (*P* = 0.002, WMD = −19.57 [−32.11, −7.02]; heterogeneity: *P* = 0.02, *I*^2^ = 66%, random-effect model, Fig. 15).

**Figure 16:**
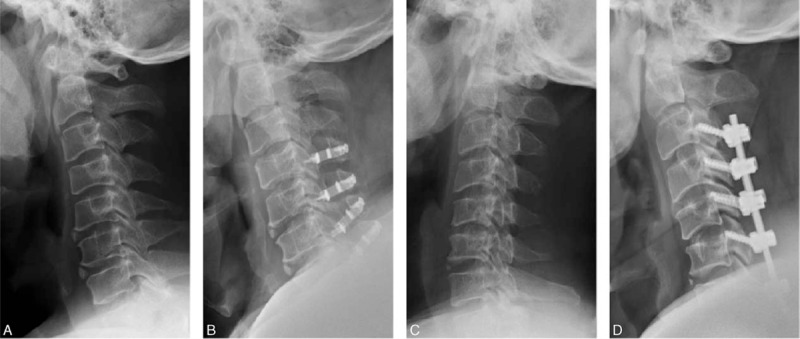
X-ray radiographs of the patients. (1A) Cervical laminoplasty preoperatively; (1B) cervical laminoplasty 1 year after operation. (2A) Cervical laminectomy and fusion preoperatively; (2B) cervical laminectomy and fusion 1 year after operation.

X-ray radiographs of the patients treated with laminoplasty or laminectomy followed by fusion were shown in Fig. 16.

### 3.6 Publication bias

Assessment of publication bias for all included studies was performed by the funnel plot on visual inspection, Egger's linear regression test, Begg's rank correlation test, and trim and fill method. The funnel plot did not indicate any publication bias in operation time (Begg, *P* = 1.000; Egger, *P* = 0.889), blood loss (Begg, *P* = 0.462; Egger, *P* = 0.573), preoperative JOA (Begg, *P* = 0.762; Egger, *P* = 0.552), postoperative JOA (Begg, *P* = 0.225; Egger, *P* = 0.236), the C5 palsy (Begg, *P* = 0.276; Egger, *P* = 0.498), the axial pain (Begg, *P* = 1.000; Egger, *P* = 0.872), total complications (Begg, *P* = 0.230; Egger, *P* = 0.537), postoperative CCI (Begg, *P* = 1.000; Egger, *P* = 0.106), preoperative VAS (Begg, *P* = 0.806; Egger, *P* = 0.859), preoperative cervical lordosis (Begg, *P* = 0.296; Egger, *P* = 0.272), and postoperative cervical lordosis (Begg, *P* = 1.000; Egger, *P* = 0.530). The funnel plots demonstrated a slight asymmetry in JOA scores improvement rate (Begg, *P* = 0.583; Egger, *P* = 0.051), preoperative CCI (Begg, *P* = 0.024; Egger, *P* = 0.154), and postoperative VAS (Begg, *P* = 0.221; Egger, *P* = 0.019). But the trim and fill method showed no study missed in JOA scores improvement rate, 3 studies missed in preoperative CCI, and 3 studies missed postoperative VAS, which indicated a reliable analysis.

## 4 Discussion

Surgical treatment of multilevel cervical compressive myelopathy remains controversial and challenging. Laminoplasty and laminectomy followed by fusion are 2 of the most commonly performed posterior procedures for the treatment of multilevel cervical compressive myelopathy.^[5–8]^ Several articles have previously addressed decision making in the treatment of multilevel cervical compressive myelopathy. Yoon et al^[1]^ conducted a systematic review comparing laminoplasty and laminectomy followed by fusion, but could not give pooled data. Lee et al^[3]^ conducted a meta-analysis comparing laminoplasty and laminectomy followed by fusion, but without pooled data about operation and complication. Therefore, we designed this meta-analysis to analysis data about clinical outcome, operation, and complication.

JOA score and VAS were often used to evaluate the improvement of nerve function.^[7–14]^ The pooled data showed that there were significant postoperative increased JOA score and VAS between 2 groups. However, the difference in preoperative and postoperative JOA score, JOA score improvement rate, preoperative, and postoperative VAS were not statistically significant. Hence, both the 2 techniques can have sufficient decompression and nerve improvement. From a biomechanical point of view, laminoplasty and laminectomy followed by fusion were similar.^[15]^ In both techniques, the muscles were widely dissected, ligamentous structures transected, and the lamina were removed or opened.^[16]^ As both the 2 surgical approaches removed spinal cord compression, symptoms were improved. So both techniques were effective.

The C2–C7 Cobb angle and CCI were often used to evaluate cervical lordosis.^[17–19]^ This study showed that there were no significant difference among the 2 groups in both the C2–C7 Cobb angle and CCI. Therefore, postoperative cervical lordosis was similar. As the muscles were widely dissected and ligamentous structures transected, both techniques tended to lose cervical lordosis to greater or lesser extent. When choosing surgery technique before the operation, we should evaluate cervical lordosis of patient with multilevel cervical compressive myelopathy. We should choose laminectomy followed by fusion if the patient with severe cervical kyphotic deformity in preoperation. However, both techniques were advisable if the patient without cervical kyphotic deformity in preoperation.

Postoperative complications were selected for analysis. As 2 of the most important complications of posterior procedures, C5 palsy and axial pain were also selected for analysis.^[20–25]^ There was no significant difference in the total complications and axial pain between 2 groups. However, compared with laminectomy followed by fusion, laminoplasty showed fewer C5 palsy. C5 palsy is a notorious complication following the cervical posterior approach. Tethering of the nerve root is considered a risk factor of C5 palsy. Spinal cord drifts after posterior decompression. As C5 nerve root is shorter than other nerve root and the C5 level is generally cervical lordosis vertices, spinal cord drift back sharpest, so C5 nerve root palsy occurred most often. In the laminoplasty group, the limited inclination angle of vertebral plate makes spinal cord drift limited.^[7]^ Therefore, laminoplasty showed fewer C5 palsy. However, increased cervical lordosis and more decompression in laminectomy followed by the fusion group increase the tethering effect of the nerve roots.^[26–29]^ Hence, improving the cervical lordosis and more decompression may lead to a high incidence of C5 palsy.

Operation time and blood loss were important factors for assessing surgical trauma.^[30]^ This study showed that there was no significant difference in the blood loss but significant difference in the operation time between 2 groups, which means that the surgical trauma is smaller in laminoplasty than in laminectomy followed by fusion.

### 4.1 Study limitations

There were several limitations in this study. First, the qualified studies included Chinese and English studies. Due to the patient's physical difference may lead to different curative effect. Second, laminoplasty had different techniques, such as open door and French door and these differences were not considered. Third, follow-up time varied between the studies and thus may have influenced our results. Finally, only one of the studies included in the meta-analysis was randomized controlled trial (RCT).

## 5 Conclusions

Both laminoplasty and laminectomy followed by fusion may result in clinical improvement and a similar loss of lordosis. As compared with laminectomy followed by fusion, expansive laminoplasty showed shorter operation time and fewer C5 palsy. To provide objective data on the clinical results of both procedures, a well-designed and prospective RCT should be performed in the future.
